# The Efficacy of Low-Level Laser Therapy on the Healing of Oral Wounds: A Systematic Review

**DOI:** 10.7759/cureus.70832

**Published:** 2024-10-04

**Authors:** Lipika Gopal, Pooja Palwankar, Nipun Dhalla

**Affiliations:** 1 Periodontology, Manav Rachna Dental College, School of Dental Sciences, Manav Rachna International Institute of Research and Studies, Faridabad, IND

**Keywords:** biostimulation, low-level laser therapy, oral soft tissue healing, oral wound healing, photobiomodulation

## Abstract

Wound healing is an intricate process involving biological alterations in cellular integrity. Low-level laser therapy (LLLT) includes special features that speed up tissue regeneration and wound healing. The objective of this review is to examine the outcomes of photobiomodulation therapy in terms of healing. A search strategy was prepared using MeSH terms and Boolean operators. The initial search was limited to randomized controlled trials in the English language from January 2014 until January 2024. An electronic search of the National Library of Medicine (NLM catalog), Google Scholar, and Scopus was conducted using Preferred Reporting Items for Systematic Review and Meta-Analyses guidelines. The studies used LLLT of various wavelengths and parameters on oral wound healing. A total of 54 records were identified through database searching. In total, 14 studies were included according to the inclusion criteria. Ten studies examined the effects of LLLT in post-surgical hard and soft tissue healing in humans. Four animal studies observed the efficacy of photobiomodulation on the healing of bone tissue. All investigations found a substantial contrast between control and laser groups concerning wound epithelialization. When used as an adjunct or a substitute, LLLT has advantages in terms of pain and inflammation by boosting acetylcholine esterase synaptic activity, beta-endorphin synthesis, and serotonin production. By promoting the growth of osteoblasts and fibroblasts, LLLT can enhance the production of new bone at an early stage. Hence, further studies and meta-analyses are required for a better understanding of the mechanism and to confirm the efficacy with different parameters.

## Introduction and background

The laser light is considered a therapeutic tool with analgesic, anti-inflammatory, and healing properties. At the Hughes Research Laboratory in California, on May 16, 1960, Theodore Maiman ignited the first laser by shining a powerful flash lamp on a ruby rod with surfaces coated in silver. Mester et al. originally described the usage of lasers in 1971 [1]. In 1989, we saw the introduction of modified ophthalmic neodymium:yttrium aluminum garnet (Nd:YAG) laser into dentistry [2]. The number of laser therapy procedures performed has grown significantly over the past few decades, starting with its initial dental application. During the past 10 years, laser dentistry has reached its pinnacle in both clinical and research settings. The acronym LASER stands for light amplification by stimulated emission of radiation [2]. It is a piece of equipment that creates a non-divergent monochromatic light beam from light with varying frequencies. This light beam, which produces electromagnetic radiation with a consistent wavelength, phase, and polarization, is said to be collimated, intense, and coherent. Lasers can be identified by their wavelengths and the tissue they act on. There are two types of lasers in terms of tissue interaction, namely, soft tissue lasers and hard tissue lasers. Holmium, Nd:YAG, argon, diode, and carbon dioxide (CO_2_) lasers are examples of soft tissue lasers. Erbium:yttrium scandium-gallium-garnet (Er:YSGG) and erbium:YAG (Er:YAG) are examples of hard tissue lasers [3]. The laser is affected by the wavelength and dose of the laser beam. Lasers with less than 90 mW of low-energy power are classified as low dose. These lasers are thought to be a kind of intense, focused light treatment as they emit the lowest energy level [4].

Low-level laser therapy (LLLT) is advised for tissue biostimulation at low dosages. Consequently, phototherapy (low-power, coherent monochromatic light) is used in LLLT and has been demonstrated to have effects on wound biostimulation, fibroblast proliferation, and collagen synthesis [3]. It is a non-invasive treatment that features an energy density of 0.04-50 J/cm2, a wavelength range of 600-1,100 nm, and an output power of 1-500 mW [4]. It is preferred over conventional lasers due to its high degree of healing capabilities and other therapeutic applications, including reduced inflammation, edema, and discomfort following surgery.

Different studies may use varying wavelengths, dosages, and treatment durations. A review can help identify the most effective parameters for LLLT in oral wound healing. Establishing standardized protocols based on reviewed evidence can lead to more consistent and reliable treatment outcomes. The review can point out gaps in the evidence or potential problems with the current studies. This identification can inform future study directions and experimental designs. Scientists can suggest new research topics or hypotheses for additional study by analyzing recent results.

Scope of the review

This systematic review includes studies focussed on analyzing the effect of photobiostimulation on oral wound healing either used independently or as an adjunct to post-surgical procedures. Hard and soft tissue healing is emphasized in human and animal models in this review.

Objective

The current literature on LLLT has conflicting results on its effect when used alone or as an adjunct on the healing of oral wounds [2]. The goal of this review is to determine if LLLT enhances wound healing when compared to sites/regions without LLLT post-surgery.

## Review

Methodology

**Figure 1:**
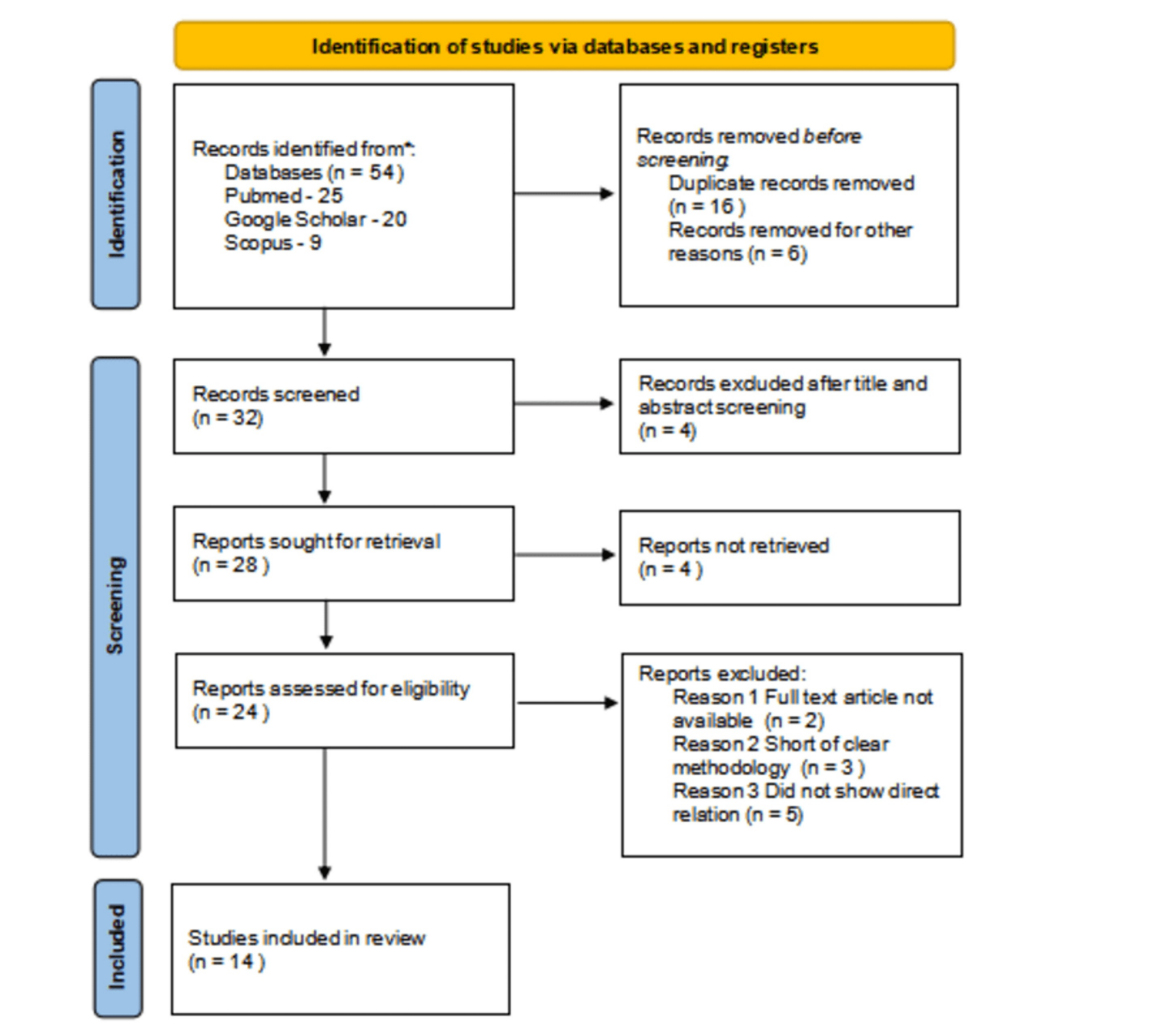
Flowchart of the study selection process based on Preferred Reporting Items for Systematic Reviews and Meta-Analyses (PRISMA) guidelines.

The study protocol was formulated based on the Preferred Reporting Items for Systematic Reviews and Meta-Analyses (PRISMA) statement (Figure 1).

The protocol was put up for registration with receipt number 579673 in the International Prospective Register of Systematic Reviews (PROSPERO). The methods and protocol of the review were established before conducting the actual review. A meta-analysis was planned to be conducted if found appropriate. There were no deviations from the planned protocol as set up in PROSPERO.

The PICOS (Patient/Population, Intervention, Comparison, Outcome, Study) design served as a foundation for the development of eligibility requirements for systematic reviews. Based on the PICOS criteria, the research question was framed as “Does low-level laser therapy help to enhance wound healing post-surgery?” PICOS was defined as follows: P: Population was taken as humans/whole animals; I: Intervention used was LLLT; C: comparison done with placebo or laser with different parameters; O: Outcome based on pain assessment and soft/hard tissue healing; and S: Studies included were randomized controlled trials and comparative studies. Periodical duplications were not included. Additionally, surveys containing unextractable study outcome data were removed. Lastly, we determined that the studies were ineligible for inclusion if the complete text was not available.

Search Strategy

A systematic review was conducted to analyze the effect of LLLT on oral wound healing. The literature was searched from January 2015 to June 2024 from databases such as NLM catalog, Google Scholar, and Scopus using keywords such as photobiomodulation, oral wound healing, LLLT biostimulation, and oral soft tissue healing. The search strategy was done using Boolean operators such as Low-Level Laser Therapy AND wound healing, Photobiomodulation OR Low-Level Laser Therapy, Biostimulation OR Low-Level Laser Therapy for all the databases.

Inclusion Criteria

The review included studies published between January 2015 and June 2024 that established laser settings, such as treatment duration and power output. Studies in which the ailment being treated was made explicit were considered for inclusion. The studies needed to describe the type of laser utilized and its specified wavelength mentioned. Studies that were either published or had full preprints available were considered for inclusion. 

Exclusion Criteria

Studies published in languages other than English were excluded. Repeated studies in the databases were also excluded. Studies in which there was no data interpretation or display of study outcomes were excluded. Finally, studies with missing information about intended results were excluded.

Results

In this review, 54 studies were initially observed in online databases (PubMed, 25; Google Scholar, 20; and Scopus, 9). After the first phase, 22 records were excluded; 16 duplicate and six case reports/series were removed. Then, 32 clinical/laboratory studies were assessed, of which four records were removed after the title and abstract screening. Subsequently, 28 records were sought for retrieval, of which four records were not retrieved. The 24 studies were assessed for eligibility, and based on the exclusion and inclusion criteria, 10 studies did not have full text, six studies were short of clear methodology, and four did not show a relation. After a thorough assessment of numerous articles, 14 clinical studies met our inclusion criteria.

Data Extraction

Two reviewers searched for the data using the search strategy from each paper. We obtained clinical information, which encompassed demographic information and research design, including methodology, laser application parameters, follow-up, and primary results. The study results were tabulated under the following headings: author and year, study design, population, type of laser, laser specification, and conclusions (Table 1).

**Table 1:** Characteristics of studies included in the review.

Author and year	Study design	Population	Type of laser	Laser specification	Conclusions
Pathak et al. [5] 2024	A single-blinded, randomized clinical trial	32 individuals who needed extraction of impacted third molars	940 nm diode laser	Photobiomodulation therapy at intraoral and extraoral points preoperatively, postoperatively, and two days after the procedure	Pain and mouth opening was reduced in subjects exposed to photobiomodulation wherein the individuals showed improvement on the second and seventh postoperative days in terms of mouth opening and pain, respectively
Misra et al. [6] 2023	Randomized controlled trial	40 subjects with chronic periodontitis and attachment loss bilaterally, pocket depths of 5 mm, and horizontal bone loss shown radiographically	890 nm diode laser	At baseline, the power output of 1.5 W in non-contact mode, post-flap surgery third and seventh day for 30 seconds	Significant improvement in healing post-flap surgery was noted after photobiomodulation. The Landry Wound Healing Index showed the test group had significantly better wound healing than the control group (3.5 ± 0.67 vs. 2.5 ± 0.50, p < 0.001, S) on the seventh day. The Landry Wound Healing Index values in the second week were 4 ± 0.49 and 3 ± 0.75, indicating significantly better healing in the test group when compared with the control group (p < 0.001)
Camolesi et al. [7] 2023	Randomized clinical trial	40 dental implants placed in 13 patients	diode laser of 630 and 808 nm)	Photobiomodulation at baseline and seventh day postoperatively with total energy density of 33.3 J/cm^2^	The therapy significantly reduced inflammation and enhanced healing in bone and mucosal tissue with 808 nm and 630 nm, respectively. The PBM− group had just two implants (18.2%) with a maximum healing index (HI = 5), while the PBM+ group had nine implants (45%) with the same index
Shah et al. [8] 2021	Parallel-group, double-blind, randomized controlled trial	40 subjects with periapical lesions	660 nm diode laser	LLLT was applied at the peri-apex for one minute for each session with 660 nm Duolase laser with a power setting of 100 mW	The patient’s pain level decreased as a result of the use of LLLT during treatment. Furthermore, it was noted that the test group experienced a higher decrease in lesion size than the non-laser group which was statistically significant at three months (p = 0.014) and nine months (P = 0.026)
Reza et al. [9] 2021	Randomized clinical trial	21 patients	810 nm and 660 nm diode laser	Energy density of 6 J/cm^2^	Enhanced healing in terms of pain in the laser test group at all periods assessed (p < 0.05). The Wilcoxon test revealed that the laser side had significantly improved wound healing scores at three (p < 0.001), seven (p < 0.001), and 14 (p = 0.03) days compared to the placebo group. A paired t-test revealed that the mean pain index on the laser side was significantly lesser than that on the placebo side after 12 hours (p < 0.008), 24 hours (p < 0.04), 48 hours (p < 0.008), and 72 hours (p < 0.02)
Rigonato-Oliveira et al. [10] 2019	Experimental design	House Dust Mite (HDM)	660 nm diode laser	Power of 30 mW on an area of 0.045 J/cm^2^, and 1, 3, 5, and 7 Jof energy density in the LLLT mode. The protocol consisted of thrice weekly irradiation for five weeks	It has been demonstrated that LLLT lessens the peribronchial infiltration, the local inflammatory process, and the buildup of collagen and mucus in the airways. The results are based on 10 mice per experimental group. # p < 0.001 when compared to the Basal group, Δ p < 0.01 when compared to the asthmatic group (HDM), and ns (not significant); ∗ p < 0,05 and ∗∗ p < 0,01 when compared HDM + LLLT (3J) group with different doses
Deynek et al. [11] 2019	Randomized clinical trial	Wistar rats	940 nm indium gallium arsenide phosphide laser	Power output of 0.1 W	Accelerated bone development in rats with orthopedically enlarged mid palatal sutures. Furthermore, an increased number of osteocytes (p < 0.01 osteoblasts were found on the seventh day. day lllg had significantly higher osteocyte counts than cg and hllg>
Hamzah et al. [12] 2019	Randomized clinical trial	Clinically healthy patients	940 nm, diode laser	The power output of 0.8 W	Significant reduction in pain postoperatively (p = 0.001). Also, the level of bleeding scores and discomfort was lowered. A significant difference was observed only on the fifth day (p < 0.05) postoperatively. Significant variations in postoperative discomfort levels were seen from the first day to the fifth, sixth, and seventh days
Lingamaneni et al. [13] 2018	Randomized, double-blind, split-mouth clinical study	Systemically healthy individuals requiring gingivectomy procedure	810 nm, diode laser	0.1 W of power output in continuous mode for five minutes	The subjects showed enhanced wound healing on the 14th postoperative day. This accounts for the better surface epithelialization accounted for in the study. Surface keratinization examination revealed no statistically significant differences between the stained areas of both groups at baseline, third, and seventh-day post-surgery. However, on the 14th postoperative day, healing was considerable in comparison to the controls (p = 0.001)
Noda et al. [14] 2016	Split-mouth trial	Systemically healthy Sprague-Dawley rats	650 nm, diode laser	High-frequency pulsed mode with 0.28 W power output	Bone mineral content and density improved along with the volume of bone in the laser irradiated site. There is a significant amount of osteocalcin (p = 0.04) and the number of PCNA-positive cells (p = 0.01). The mean height of freshly generated immature woven bone was greater in laser-treated areas (p = 0.24) on the seventh day
Alan et al. [15] 2016	Randomized controlled trial	Subjects with impacted third molars requiring extraction bilaterally	810 nm gallium-aluminium-arsenide diode laser	Power output of 300 mW	Pain reduced significantly on the seventh day postoperatively (p
Hamad et al. [16] 2016	Randomized clinical trial	Male rabbits	808 nm, gallium-aluminiun arsenide diode laser	0.9W power output	The sockets in the test group had enhanced bone formation and accelerated bone maturation. There was a high incidence of new bone formation and bone maturation. The volume of bone trabecule pattern and bone density was enhanced in the test group. Compared to the control sides, low-level laser-treated sides demonstrated a major decrease in visual analog pain ratings (VAS) (p < 0.05).
Khalighi, et al. [17] 2016	Randomized controlled clinical trial	40 MPDS patients	810 nm gallium-aluminum-arsenide diode source	0.5 W power output, 5 mm focal spot size	Enhanced pain reduction and there is statistically significant improvement in mouth opening in test sites. The laser group showed a substantial decrease in pain intensity in the temporalis, medial pterygoid, and lateral pterygoid muscles (p < 0.0)
Halon et al. [18] 2015	Randomized split-mouth control trial	Patients with HIV who needed extraction	Diode laser	820 nm, 200 mW, 6 J/cm^2^	Laser therapy accelerated wound healing. The vessel surface area was significantly higher in patients exposed to radiation. The differences were statistically significant for all fields (p = 0.0002)

Risk of Bias Assessment

The Physiotherapy Evidence Database PEDro presents the PEDro scale, which consists of 11 criteria for assessing clinical random sample allocation that was carried out by an evaluator. Every article that did not meet the eligibility requirements was removed, and the cause for removal was noted along with the article’s contents. Disagreements over selection were resolved via consensus-building discussions, with the option of a third person’s final judgment if needed (Table 2).

**Table 2:** Result of assessment of quality score by PEDro scale (Criteria 1-11).

Studies included	Eligibility criteria specified (1)	Subjects randomly allocated (2)	Concealed allocation (3)	Baseline similarity (4)	Subject blinded (5)	Therapist blinded (6)	Assessor blinded (7)	One key outcome obtained (8)	Treatment/control condition allocated (9)	The results of the between-group statistical comparison (10)	Point measures and measures of variability for key outcomes (11)	Total score
Pathak et al. [5] 2024	1	1	1	1	1	0	1	1	1	1	0	9
Misra et al. [6] 2023	1	1	1	1	0	0	0	1	1	1	1	8
Camolesi et al. [7] 2023	1	1	1	0	1	1	1	1	1	1	0	9
Shah et al. [8] 2021	1	1	1	1	1	1	0	0	1	1	0	8
Reza et al. [9] 2021	1	1	1	1	1	1	0	1	1	1	0	9
Rigonato-Oliveira et al. [10] 2019	1	1	1	1	0	0	0	1	1	1	0	9
Deynek et al. [11] 2019	1	1	1	0	0	0	0	1	1	1	1	7
Hamzah et al. [12] 2019	1	1	1	1	0	0	1	1	1	1	1	9
Lingamaneni et al. [13] 2018	1	1	1	1	0	0	1	1	1	1	1	9
Noda et al. [14] 2016	1	1	1	1	0	0	0	1	1	1	1	9
Alan et al. [15] 2016	1	1	1	1	0	1	1	1	0	1	1	9
Hamad et al. [16] 2016	1	1	1	1	1	0	0	1	1	1	1	9
Khalighi et al. [17] 2016	1	1	1	1	1	0	1	1	1	0	1	9
Halon et al. [18] 2015	1	1	1	1	0	0	0	1	1	1	1	8

Interpretation of Risk Assessment Bias

Of the 14 studies selected, three studies showed a lack of Criteria 4, five studies were compliant with Criteria 5 and 6, four studies were without Criteria 7, and six studies were without Criteria 11. Only one study indicated a deficit in Criteria 8, 9, and 10. The total sum of each study included in the review accounted for an inference of Good/Excellent.

Discussion

The 11 studies showed that LLLT significantly decreased postoperative pain and enhanced wound healing and three studies showed a decrease in pain and time of wound healing with no statistical significance. Pathak et al. showed the benefits of LLLT with a diode laser of 940 nm wavelength following surgery in which 32 subjects requiring extraction of impacted third molars were divided into the control and test groups. The study group received LLLT at intraoral and extraoral selected locations before, during, and two days after the procedure. The test group subjects had less postoperative pain, but the reduction in swelling was statistically non-significant [5]. Misra et al. aimed to clinically examine wound healing and biochemical examination of gingival crevicular fluid (GCF) containing mediators of inflammation with open flap debridement treated with LLLT. In this study, 120 control and test sites were selected, the wound healing index was recorded in the first and second week, and GCF inflammatory mediators were evaluated at periodic intervals. The results recorded a decrease in inflammatory markers and high levels of osteoprotegerin levels in the test group. The Landry Wound Healing Index values in the first and second weeks also demonstrated statistically significant better healing in the test group [6]. The randomized clinical experiment Camolesi et al. [7] aimed to determine the effect of photobiomodulation on inflammation, implant stability, and healing postoperatively. Dental implants selected in the study were divided into test and control groups randomly and the test sites were exposed to LLLT immediately after surgery and on the seventh day post-surgery. They observed that over time no variations were seen in the implant stability values between the control and test groups. The photobiomodulation- group showed the highest healing index (HI = 5) in only two implants (18.2%), while the photobiomodulation+ group showed nine implants (45%) with the same value (p < 0.0001). Using logistic regression, it was determined that not using the laser in the PBM group increased the likelihood of presenting inflammation by 4.333 times (IC95% = 1.150-16.323; p = 0.030)[7]. A study by Shah et al. showed that at the three- and nine-month follow-up, significant variations in the decrease of periapical lesions were seen. In Group I, which underwent LLLT together with traditional root canal therapy (RCT), the healing process was more successful. Although they were statistically insignificant, Group I postoperative pain values were lower than Group II [8]. The data gathered in the present research indicates that photobiomodulation can be suggested as a supplement to traditional medical treatment of acute lung injury caused by sepsis because of its ability to stop the advancement of respiratory parenchyma injury by reducing the inflammation-induced condition and shorten the recovery time of patients with APL caused by sepsis. [10]. In both human and animal research, LLLT was presented as a primary or adjuvant therapy agent with positive outcomes. We investigated the effectiveness of photobiomodulation on oral wound healing. According to Hamad et al. [16], during the first three postoperative days, the test sites of low-level laser irradiation sides showed a drastic decrease in pain scales (Visual Analog Scale (VAS)) (P<0.05). The VAS scores recorded for the laser-treated sides were 4.46, 4.00, and 3.35, while for the control the scores were 6.58, 5.82, and 5.17. In this study, the test sites treated with laser were compared to the control sides. On the first and third postoperative days, there was a substantial reduction in edema and trismus (p < 0.05). Unexpectedly, lasers are now a standard component of our treatment plans. This review has discovered the advantages of using LLLT alone as well as an adjunct. There were many different subjects because the studies comprised human and animal investigations.

In a study by Mohajerani et al., there was less trismus, and between days three and seven post-extraction, there was also less inflammation [19]. Photobiomodulation therapy (PBMT) helps reduce postoperative pain and inflammation. The findings indicated that there might be a connection between a drop in inflammatory processes following PBMT and a drop in salivary secretory IgA levels [20]. It has been claimed that LLLT can be used as an adjuvant therapy in addition to standard treatments to enhance the process of wound healing and increase patient satisfaction. Numerous clinical studies have attempted to measure the effectiveness of photobiomodulation in aiding healing and pain reduction, but their findings have been conflicting [21,22]. According to Lingamaneni et al., patients in overall good condition showed improved surface epithelization following gingivectomy procedures when LLLT was used [13]. Rats with elevated osteoblast levels also showed favorable effects on bone production, according to Deynek et al [11]. In their investigation of rabbits, Hamad et al. [16] discovered that LLLT inserted into post-extraction sockets accelerated the development of primary to secondary bone. Currently, one of the fields of biomedical science with the quickest growth is photobiomodulation. Laser and LED light can speed up healing, ATP generation, and cell differentiation [23]. This review found no unanimity on how low or high-energy density affects epithelized or residual wound areas. Similar meta-analyses yielded conflicting results, with some indicating that low-energy density (< 4 J/cm^2^) led to superior healing outcomes [24,25]. Lasers with an energy density ranging from 0.5 J/cm^2^ to 16 J/cm^2^ may be a source of disagreement in research on postoperative pain control with an average of 8 J/cm^2^. According to one idea, the pain-relieving effects were dependent on the dosage of light waves until the limit was reached, at which point they became ineffective due to radiation saturation [26,27].

In this systematic review, LLLT has an inherent propensity to hasten wound healing and reduce postoperative pain which makes it a potentially useful technique. To offer more robust proof, however, more research with bigger samples and placebo/active comparators is required.

Limitations

Despite the findings provided in all the included studies, the limitation of this study is the exclusion of information that may exist outside of the Scopus, PubMed, and Google Scholar databases. While these platforms are well-known for their fullness and academic representation, probably some relevant works in dental lasers were not fully covered in these sources. This is a partial picture of the progression of trends in dental lasers across the investigated time. While many studies report positive outcomes, the documentation on the efficacy of LLLT in oral wound healing is somewhat mixed. Some systematic reviews and meta-analyses have shown significant benefits, while others suggest more research is needed to confirm its effectiveness in specific conditions. Due to the variations in wavelength, energy density, and time period, a meta-analysis was not possible.

## Conclusions

Even if the quantity of research supporting the application of LLLT is rising in promoting wound healing and managing associated symptoms, including pain and inflammation, further well-designed clinical trials are needed to provide precise standards and procedures for its appropriate use in various oral conditions. There is still much to learn about the mechanisms, establishing the curative opening, and correctly using these biological occurrences to achieve therapy goals.

The clinical significance of LLLT in promoting oral wound healing stems from its potential to expedite healing, reduce pain and inflammation, and enhance overall patient outcomes. As research advances, LLLT may become an essential component of routine practices in oral medicine and dentistry.
