# Correction: The Efficacy of Low-Level Laser Therapy on the Healing of Oral Wounds: A Systematic Review

**DOI:** 10.7759/cureus.c319

**Published:** 2025-09-24

**Authors:** Lipika Gopal, Pooja Palwankar, Nipun Dhalla

**Affiliations:** 1 Periodontology, Manav Rachna Dental College, School of Dental Sciences, Manav Rachna International Institute of Research and Studies, Faridabad, IND

This article has been corrected to fix the following typos in the first paragraph of the Results section:

- 25 articles were found in PubMed, not the NLM catalog
- Four articles, not five, were removed after the title and abstract screening

